# Seroprevalence of *Helicobacter pylori* in Korea: A multicenter, nationwide study conducted in 2015 and 2016

**DOI:** 10.1111/hel.12463

**Published:** 2018-01-18

**Authors:** Jeong Hoon Lee, Kee Don Choi, Hwoon‐Yong Jung, Gwang Ho Baik, Jong Kyu Park, Sung Soo Kim, Byung‐Wook Kim, Su Jin Hong, Hyun Lim, Cheol Min Shin, Si Hyung Lee, Seong Woo Jeon, Ji Hyun Kim, Cheol Woong Choi, Hye‐Kyung Jung, Jie‐Hyun Kim, Suck Chei Choi, Jin Woong Cho, Wan Sik Lee, Soo‐Young Na, Jae Kyu Sung, Kyung Ho Song, Jun‐Won Chung, Sung‐Cheol Yun, Korean College of Helicobacter and Upper Gastrointestinal Research

**Affiliations:** ^1^ Department of Gastroenterology Asan Medical Center University of Ulsan College of Medicine Seoul Korea; ^2^ Department of Internal Medicine Hallym University College of Medicine Chuncheon Korea; ^3^ Department of Internal Medicine Gangneung Asan Hospital University of Ulsan College of Medicine Gangneung Korea; ^4^ Department of Internal Medicine Uijongbu St. Mary's Hospital The Catholic University of Korea Uijongbu Korea; ^5^ Department of Internal Medicine Incheon St. Mary's Hospital College of Medicine The Catholic University of Korea Incheon Korea; ^6^ Department of Internal Medicine Soonchunhyang University College of Medicine Bucheon Korea; ^7^ Department of Internal Medicine Hallym University College of Medicine Anyang Korea; ^8^ Department of Internal Medicine Seoul National University Bundang Hospital Seongnam Korea; ^9^ Department of Internal Medicine Yeungnam University College of Medicine Daegu Korea; ^10^ Department of Internal Medicine School of Medicine Kyungpook National University Daegu Korea; ^11^ Department of Internal Medicine Inje University College of Medicine Busan Korea; ^12^ Department of Internal Medicine Pusan National University Yangsan Hospital Yangsan Korea; ^13^ Department of Internal Medicine College of Medicine Ewha Womans University Seoul Korea; ^14^ Department of Internal Medicine Gangnam Severance Hospital Yonsei University College of Medicine Seoul Korea; ^15^ Department of Internal Medicine and Digestive Disease Research Institute Wonkwang University School of Medicine Iksan Korea; ^16^ Department of Internal Medicine Presbyterian Medical Center Jeonju Korea; ^17^ Department of Internal Medicine Chonnam National University Medical School Gwangju Korea; ^18^ Department of Internal Medicine Jeju National University School of Medicine Jeju Korea; ^19^ Department of Internal Medicine Chungnam National University School of Medicine Daejeon Korea; ^20^ Department of Internal Medicine Konyang University College of Medicine Daejeon Korea; ^21^ Department of Internal Medicine Gachon Graduated School of Medicine Gil Medical Center Incheon Korea; ^22^ Department of Clinical Epidemiology and Biostatistics Asan Medical Center University of Ulsan College of Medicine Seoul Korea

**Keywords:** *Helicobacter pylori*, Korea, prevalence

## Abstract

**Background:**

The Korean College of *Helicobacter* and Upper Gastrointestinal Research has studied *Helicobacter pylori (H. pylori)* prevalence since 1998 and found a dynamic change in its prevalence in Korea. The aim of this study was to determine the recent *H. pylori* prevalence rate and compare it with that of previous studies according to socioeconomic variables.

**Methods:**

We planned to enroll 4920 asymptomatic Korean adults from 21 centers according to the population distribution of seven geographic areas (Seoul, Gyeonggi, Gangwon, Chungcheong, Kyungsang, Cholla, and Jeju). We centrally collected serum and tested *H. pylori* serum IgG using a chemiluminescent enzyme immunoassay.

**Results:**

We analyzed 4917 samples (4917/4920 = 99.9%) from January 2015 to December 2016. After excluding equivocal serologic results, the *H. pylori* seropositivity rate was 51.0% (2414/4734). We verified a decrease in *H. pylori* seroprevalence compared with previous studies performed in 1998, 2005, and 2011 (*P *<* *.0001). The *H. pylori* seroprevalence rate differed by area: Cholla (59.5%), Chungcheong (59.2%), Kyungsang (55.1%), Jeju (54.4%), Gangwon (49.1%), Seoul (47.4%), and Gyeonggi (44.6%). The rate was higher in those older than 40 years (38.1% in those aged 30‐39 years and 57.7% in those aged 40‐49 years) and was lower in city residents than in noncity residents at all ages.

**Conclusions:**

*Helicobacter pylori* seroprevalence in Korea is decreasing and may vary according to population characteristics. This trend should be considered to inform *H. pylori*‐related policies.

## 1 INTRODUCTION

*Helicobacter pylori* (*H. pylori*) has infected more than half of the world's population^1^ and is an important cause of gastric cancer, mucosal‐associated lymphoid tissue lymphoma, and peptic ulcer disease.^2^ Chronic infection with *H. pylori* is strongly associated with gastric cancer^3^ the highest incidence of which is observed in Korea, Mongolia, Japan, and China.^4^ Eradication of *H. pylori* has thus been attempted in China and Japan to reduce gastric cancer levels.^5^, ^6^ Accordingly, determination of the *H. pylori* prevalence of normal asymptomatic participants is crucial for the establishment of national health policies in these Eastern Asia countries. Nationwide studies of *H. pylori* prevalence were performed in Korea in 1998, 2005, and 2011.^7^, ^8^, ^9^ Although these studies obtained data from a large number of participants, they had several notable limitations, such as inconsistencies in test methods, uneven study populations, and lack of socioeconomic information. Accordingly, the Korean College of *Helicobacter* and Upper Gastrointestinal Research has newly performed a nationwide *H. pylori* prevalence study and compared its results with those of previous studies to compensate for their defects.

## 2 METHODS

### 2.1 Study population

Study participants were prospectively enrolled from 21 centers in South Korea from January 2015 to December 2016. These centers are based on seven geographic areas: Seoul (3 centers), Gyeonggi (6 centers), Gangwon (2 centers), Chungcheong (2 centers), Kyungsang (4 centers), Cholla (3 centers), and Jeju province (1 center). All participants were asymptomatic Koreans older than 16 years. Patients with gastrointestinal symptoms such as dyspepsia, regurgitation, and pain or who had history of *H. pylori* eradication, abdominal surgery, or peptic ulcer were excluded.

Informed consent was obtained, and a questionnaire on socioeconomic status was administered by a physician or nurse. The questionnaire included family history of gastric cancer, family income, education status, and habitation pattern in preschool, school, and posthigh school periods. Family history of gastric cancer was confined to parents, siblings, and children. Family income was divided into low (<US $3000 per month), medium (US $3000‐10,000 per month), and high (>US $10,000 per month). Education status was divided into low (middle school graduate or less), medium (high school graduate or university dropout), and high (university graduate or more). We investigated two aspects of habitation status, geographic area, and type of residence in terms of city or noncity during each life period.

### 2.2 Serologic evaluation

All collected serum was centrally analyzed by a single company (Seegene Medical Foundation, Seoul, Korea). The Immulite 2000^®^ *H. pylori* IgG system (Diagnostic Product Corporation, Los Angeles, CA, USA) was used to measure anti‐*H. pylori* IgG. This test consists of a solid‐phase, two‐step chemiluminescent enzyme immunoassay. The sensitivity, specificity, positive predictive value, and negative predictive value of this test are reported as 91%, 100%, 100%, and 71%, respectively.^10^ We used only positive (≥1.10 U/mL) or negative (<0.9 U/mL) results, and equivocal results (0.9‐1.09 U/mL) were excluded from the analysis.

### 2.3 Statistics

A sample size of 4920 was calculated to obtain a two‐sided 95% confidence interval with a width equal to 0.028, assuming a *H. pylori* infection rate of 55% based on a previous study.^9^ We distributed participants into seven geographic areas according to the 2013 population census. Categorical variables were analyzed by the chi‐square test. Multivariable logistic regression was used for the investigation of risk factors for *H. pylori* seropositivity. We used the Cochran‐Armitage trend test for the comparison of *H. pylori* seroprevalence among the data published in 1998,^7^ 2005,^8^ 2011,^9^ and in this study. We only analyzed the results of asymptomatic participants from previous studies. A significance level of *P *<* *.05 was applied to all analyses except for multiple comparisons. We used the Bonferroni correction to calculate the *P* values for multiple comparisons.

## 3 RESULTS

### 3.1 Seropositivity of participants and comparison with the results from 1998, 2005, and 2011

**Figure 1:**
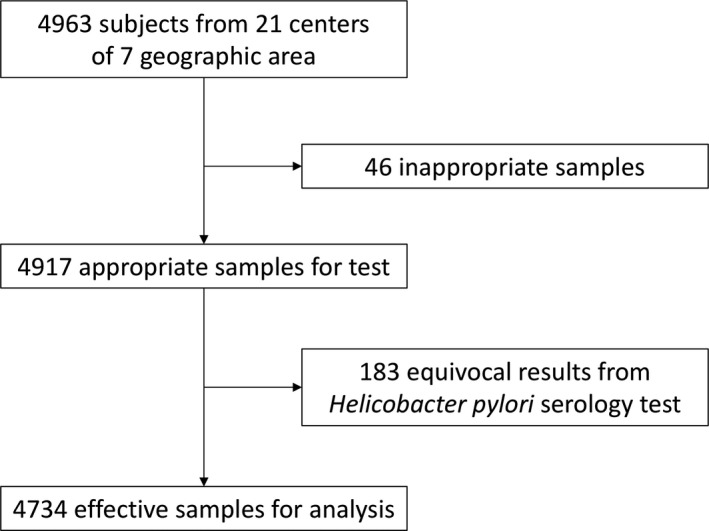
Study flowchart

We enrolled 4963 asymptomatic participants from 21 centers, and 4917 samples were found to be suitable for the *H. pylori* IgG test (Figure 1). Among these, 183 samples showed equivocal results. Thus, we acquired 4734 seropositive or seronegative results (Table 1). We found that 51.0% (2414/4734) of the study participants were seropositive.

**Figure 2:**
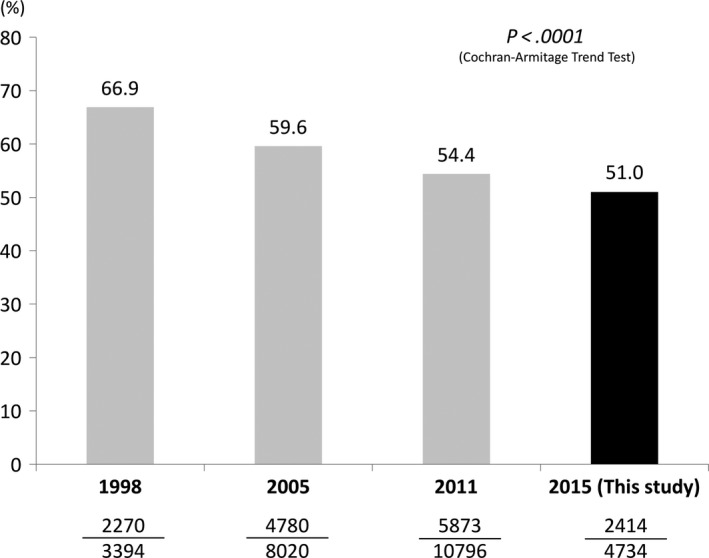
Comparison of *Helicobacter pylori* seropositivity with previous data 7, 8, 9 in asymptomatic adult participants in South Korea

We compared the *H. pylori* seropositivity of asymptomatic participants among the 1998, 2005, and 2011 studies^7^, ^8^, ^9^ and this study. The overall seropositivity of 51.0% (95% CI: 49.6‐52.4%) in this study represented a significant decrease from 66.9% (95% CI: 65.4‐68.6%) in 1998, 59.6% (95% CI: 58.5‐60.7%) in 2005, and 54.4% (95% CI: 53.5‐55.4%) in 2011 (*P *<* *.001) (Figure 2). There was thus a significant decline in seropositivity between each successive study. The arithmetical decrease rates per year were 0.81%, 0.87%, and 0.85% during 1998‐2005, 2005‐2011, and 2011‐2015, respectively.

**Table 1:** Baseline characteristics of the study population

	n	%
Total	4734	100
Sex		
Male	1937	40.9
Female	2797	59.1
Subtotal	4734	100
Age (y)		
16‐19	20	0.4
20‐29	685	14.5
30‐39	889	18.8
40‐49	1115	23.6
50‐59	1225	25.9
60‐69	603	12.7
≥70	197	4.2
Subtotal	4734	100
Geographic area		
Seoul	782	16.5
Gyeonggi	1587	33.5
Gangwon	159	3.4
Chungcheong	483	10.2
Kyungsang	1133	23.9
Cholla	511	10.8
Jeju	79	1.7
Subtotal	4734	100
Household income		
Low	1452	30.7
Medium	2655	56.2
High	621	13.1
Subtotal	4728	100
Education level		
Low	593	12.6
Medium	1371	29.0
High	2761	58.4
Subtotal	4725	100
Body mass index (kg/m^2^)		
<18.5	228	4.9
18.5‐23.0	2151	45.8
23.0‐25.0	1072	22.8
≥25.0	1242	26.5
Subtotal	4693	100
Family history of gastric cancer		
No	4362	92.2
Yes	369	7.8
Subtotal	4731	100

### 3.2 Risk factors for *H. pylori* seropositivity

**Figure 3:**
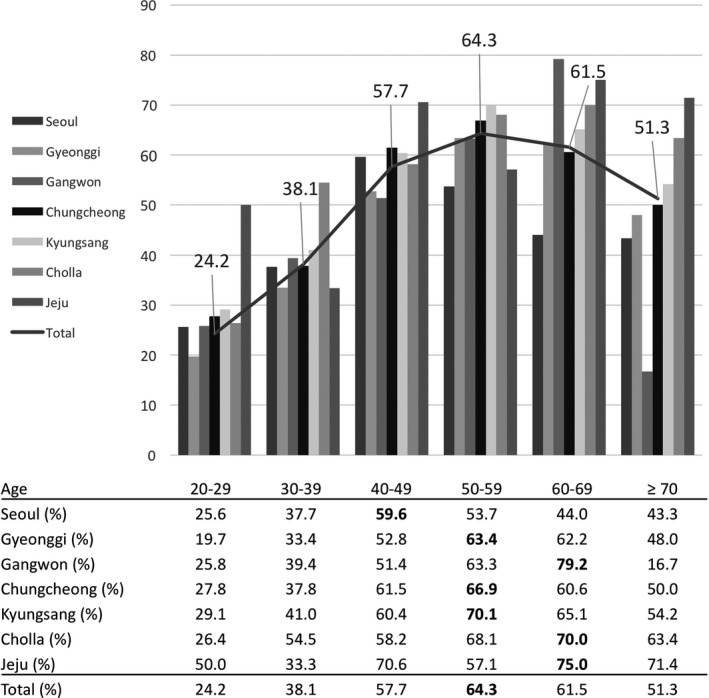
*Helicobacter pylori* seropositivity according to geographic area and age group in South Korea

Age, body mass index (BMI), geographic area, and education level were significantly associated with *H. pylori* seropositivity (Table 2). Individuals aged 20‐29 years showed the lowest *H. pylori* seropositivity (24.2%) and this increased up to 64.3% in those aged 50‐59 years (OR 4.67, 95% CI: 3.71‐5.86). A similar trend was observed according to geographic area (Figure 3). The peak prevalence group was distributed among age groups 40‐49 (Seoul), 50‐59 (Gyeonggi, Chungcheong, and Kyungsang), and 60‐69 (Gangwon, Cholla, and Jeju). Although *H. pylori* seropositivity was 32.1% (511/1594) in participants younger than 40 years, it was 60.6% (1903/3140) in those 40 or older.

**Table 2:** Risk factors for *Helicobacter pylori* seropositivity (multivariable logistic regression)

	Total	Seropositive *H. pylori*		Odds ratio	95% CI	
		N	%			
Total	4734	2414	51.0			
Sex						
Male	1937	1048	54.1	1.00	0.88	1.15
Female	2797	1366	48.8	Ref		
Age (y)						
16‐19	20	6	30.0	0.85	0.29	2.45
20‐29	685	166	24.2	Ref		
30‐39	889	339	38.1	1.85	1.47	2.31
40‐49	1115	643	57.7	3.82	3.06	4.76
50‐59	1225	788	64.3	4.67	3.71	5.86
60‐69	603	371	61.5	4.01	3.03	5.30
≥70	197	101	51.3	2.65	1.83	3.83
Geographic area						
Seoul	782	371	47.4	Ref		
Gyeonggi	1587	708	44.6	1.04	0.86	1.25
Gangwon	159	78	49.1	1.23	0.85	1.76
Chungcheong	483	286	59.2	1.33	1.03	1.72
Kyungsang	1133	624	55.1	1.46	1.20	1.77
Cholla	511	304	59.5	1.52	1.19	1.94
Jeju	79	43	54.4	1.63	0.99	2.68
Household income						
Low	1452	778	53.6	0.92	0.73	1.15
Medium	2655	1306	49.2	0.92	0.76	1.11
High	621	327	52.7	Ref		
Education level						
Low	593	368	62.1	1.21	0.95	1.56
Medium	1371	800	58.4	1.23	1.06	1.42
High	2761	1243	45.0	Ref		
Body mass index (kg/m^2^)						
<18.5	228	78	34.2	0.91	0.67	1.23
18.5‐23.0	2151	1012	47.0	Ref		
23.0‐25.0	1072	591	55.1	1.14	0.97	1.34
≥25.0	1242	707	56.9	1.17	1.00	1.37
Family history of gastric cancer						
No	4362	2199	50.4	Ref		
Yes	369	214	58.0	1.08	0.86	1.35

The multivariable logistic regression included 4681 individuals.

Participants with a high BMI (≥25.0 kg/m^2^) showed higher *H. pylori* seropositivity than those with a relatively normal BMI (18.5‐23.0 kg/m^2^) (OR 1.17, 95% CI: 1.00‐1.37).

**Figure 4:**
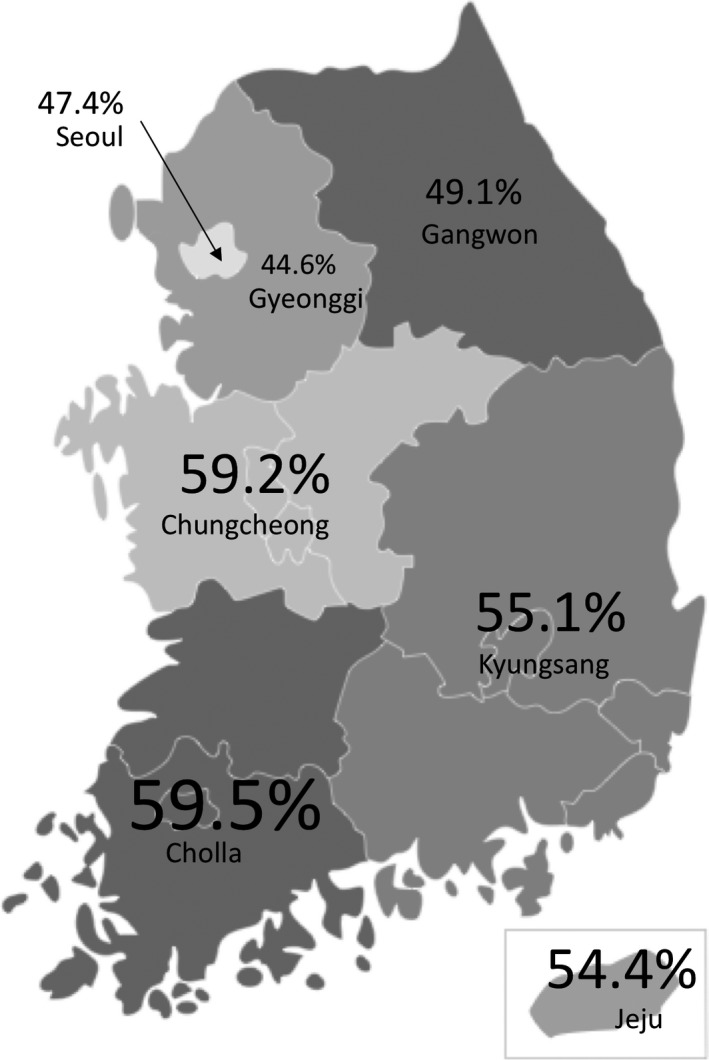
*Helicobacter pylori* seropositivity in seven geographic areas in South Korea

Chungcheong, Kyungsang, and Cholla, all located in the southern part of South Korea, showed higher *H. pylori* seropositivity than Seoul (Figure 4). *H. pylori* seropositivity rates were below 50% in Seoul and the adjacent provinces (Gyeonggi and Gangwon).

Participants who had a medium education level showed a 23% increase in *H. pylori* seropositivity compared with those with a high education level (OR 1.23, 95% CI: 1.06‐1.42). Sex, household income, and family history of gastric cancer had no influence on *H. pylori* seropositivity in multivariable logistic regression analysis.

### 3.3 Impact of habitation according to life period on *H. pylori* seropositivity

We investigated *H. pylori* seropositivity according to habitation type (city vs noncity) and life period (Table 3). Participants who lived in non‐city–city–city circumstances according to life period showed higher *H. pylori* seropositivity than those who lived in city–city–city circumstances (58.4% vs 46.6%, *P *<* *.001). Additionally, participants who lived in city circumstances throughout life showed lower *H. pylori* seropositivity than those who lived in noncity circumstances throughout life (46.6% vs 62.8%, *P *<* *.001) and showed lower *H. pylori* seropositivity than those with non‐city–non‐city–city circumstances throughout life (46.6% vs 56.9%, *P *<* *.001).

**Table 3:** *Helicobacter pylori* seropositivity according to habitation type and life period

Group	Preschool	School‐age	After high school	N	%
1^a^	City	City	City	1381/2946	46.6
2^a^	Noncity	City	City	283/485	58.4
3^a^	Noncity	Noncity	City	428/752	56.9
4^a^	Noncity	Noncity	Noncity	290/462	62.8
5	City	City	Noncity	16/30	53.3
6	City	Noncity	City	5/16	31.3
7	Noncity	City	Noncity	4/6	66.7
8	City	Noncity	Noncity	3/5	60.0

a We compared groups 1‐4 by considering the relatively large participant number. A *P* value less than .008 was considered significant due to multiple comparisons.

Group 1 vs 2, *P *<* *.001; group 1 vs 3, *P *<* *.001; group 1 vs 4, *P *<* *.001; group 2 vs 3, *P *=* *.618; group 2 vs 4, *P *=* *.164; group 3 vs 4, *P *=* *.044.

## 4 DISCUSSION

The *H. pylori* seropositivity of Korea in 2015 and 2016 was 51.0%, and we confirmed a decrease in *H. pylori* seropositivity compared with previous studies (from 66.9% in 1998 to 51.0% in 2015). In general, *H. pylori* is more prevalent in developing countries and less prevalent in developed countries. Our result agrees with the development status of Korea, whose gross domestic products per capita were $12,000 in 2000 and $27,000 in 2015.^11^

We found decreased *H. pylori* seropositivity, particularly in those aged 30‐39 years, compared with the three previous Korean nationwide studies. Previous studies reported seropositivity rates in those aged 30‐39 years of 74%, 49%, and 42% in 1998, 2005, and 2011, respectively. Considering that prevalence in less developed regions may reach 70% or higher, compared with 40% or less in more developed regions,^12^ our result (38.1%) is the first to be below 40% in those aged 30‐39 years. In addition, there was a large increase in seropositivity from those aged 30‐39 years to those aged 40‐49 years in our present study series. However, the biggest increase was shown from those aged 20‐29 years to those aged 30‐39 years in previous Korean studies.

*Helicobacter pylori* infection and gastric cancer are endemic to Japan and China, as well as Korea.^1^, ^13^ Japan has shown a decrease in *H. pylori* prevalence, similar to Korea. In several birth cohort studies from Japan, Japanese individuals born before 1950 showed a *H. pylori* prevalence rate of 80%‐90%, but those born during the 1980s showed a *H. pylori* prevalence of 10%‐20%.^14^, ^15^ Recent Japanese studies reported that the *H. pylori* seropositivity in junior high school students was only 3%‐5%.^16^, ^17^ Although we did not analyze the birth cohort effect, our lowest seroprevalence rate was 24% in those aged between 20 and 29 years. Also, the highest prevalence rate was observed in those aged between 50 and 59 years. These trends were shown across all seven geographic areas in South Korea. *H. pylori* seroprevalence data from China have also shown a decrease, from 60% in 1983 to 45% in 2010.^18^, ^19^, ^20^ Korea might show lower prevalence in the future with improved general hygiene, economic development, and a sustained decrease in prevalence among generations.

Although Korea is a small and high internal migration country, we anticipated a geographic difference in *H. pylori* seropositivity. For the first time among studies of this nature, we distributed the study population among seven geographic areas to adapt to the population census result for *H. pylori* prevalence in Korea. We found that the capital region (Seoul and Gyeonggi) showed lower *H. pylori* seropositivity than other areas. This trend was also reported in a previous Korean study in 2011.^9^ Interestingly, as the distance from the capital region increased, so did the odds ratio of *H. pylori* prevalence. This may be due to urbanization levels, and we indirectly analyzed this effect according to life period. After the exclusion of groups with a small sample size listed in Table 3, participants who lived in a city throughout their life showed the lowest *H. pylori* seropositivity. Consistently, a recent Chinese study reported that urban populations had lower rates of *H. pylori* infection than rural populations.^20^

In our study, we observed that subjects with higher education level tended to show lower *H*. pylori prevalence. Considering the increasing proportion of city residence after high school (63.9%, 86.7%, and 96.2% in low, medium, and high education level, respectively) as seen in our study population, this result may be explained by the effect of urbanization.

*Helicobacter pylori* was more prevalent in obese patients (BMI ≥25.0 kg/m^2^) than in those with a normal BMI range (OR 1.17, 95% CI: 1.00‐1.37). However, other BMI groups showed no difference compared with the normal BMI group. And the association between obesity and *H. pylori* infection is controversial, and the causality of these associations has not been proven.^2^, ^21^, ^22^ We also investigated the correlation between BMI and residence style after high school (city vs noncity) but observed no significant correlation between the two (data not shown).

We found no sex differences in the prevalence of *H. pylori* (OR 1.00, 95% CI: 0.88‐1.15), but a recent meta‐analysis of 169 studies reported that male sex was associated with a higher prevalence of *H. pylori* (OR 1.12, 95% CI: 1.09‐1.15).^23^ Our current study was not designed to determine sex differences and several confounding factors were not assessed (such as smoking history, urinary tract infection history in women, and sex hormones).

Our study had some limitations of note. First, there may have been a selection bias. We enrolled asymptomatic participants from tertiary hospitals or their health screening centers. Thus, we may have enrolled more participants with a higher socioeconomic status. Second, there could have been a recall bias. We considered the habitation status of our study patients but this is a relatively subjective parameter and might be recalled incorrectly. Third, although this was a prospective study, the enrollment period was relatively long. However, most participants (91%) were enrolled in 2015.

In conclusion, our current multicenter, nationwide study found a decrease in *H. pylori* seroprevalence in South Korea and a difference in the seroprevalence rate according to geographic area and habitation type. Our findings could be useful as future baseline data or to inform *H. pylori*‐related policies in Korea.

## DISCLOSURES OF INTERESTS

All authors declare no conflicts of interest.
